# Effectiveness of Anapana, Body scan and Metta meditation techniques on chronic neck and shoulder region pain and disability in adult patients in Sri Lanka: study protocol for a cluster clinic-level randomised controlled trial

**DOI:** 10.1186/s13063-022-06873-x

**Published:** 2022-11-15

**Authors:** Aranjan Lionel Karunanayake, Emma Solomon-Moore, Nikki Coghill

**Affiliations:** 1 Department of Anatomy, Faculty of Medicine, University of Kelaniya, Kelaniya, Sri Lanka; 2 Department for Health, Faculty of Humanities and Social Sciences, University of Bath, Claverton Down, Bath, BA2 7AY UK

**Keywords:** Neck pain, Meditation, Randomised controlled, Anapana, Body scan, Metta

## Abstract

**Background:**

Chronic neck and shoulder region pain affects many people around the world. This study aims to compare the effectiveness of three 8-week meditation training programmes (each using a different meditation technique: Anapana, Body scan or Metta) on pain and disability in a patient population affected with chronic neck and shoulder region pain, with a usual care control group and with each other.

**Methods:**

This four-arm parallel clinic-level randomised controlled trial will be conducted with male and female patients aged 18–65 years, who are affected with chronic neck and shoulder region pain, and who attend one of four clinics held on four different days of the week in a single medical centre in the Colombo North region, Sri Lanka. Clinics will be considered as clusters and randomly allocated to intervention and control arms.

Data will be collected using validated questionnaires, clinical examinations and focus groups. To compare primary (differences in changes in pain (Numeric Pain Rating Scale) at 8 weeks) and secondary (differences in changes in pain, physical disability, range of movement and quality of life (SF-36) at 4 and 12 weeks) outcomes between groups, a two-way ANOVA will be used if data are normally distributed. If data are not normally distributed, a nonparametric equivalent (Kruskal-Wallis) will be used. Focus group transcriptions will be thematically analysed using the Richie and Spencer model of qualitative data analysis.

**Discussion:**

This is a four-arm trial which describes how three different 8-week meditation technique (Anapana, Body Scan, Metta) interventions will be implemented with adult patients affected with chronic neck and shoulder region pain. The effectiveness of each meditation intervention on the pain, physical and psychosocial disabilities of patients will be compared between groups and with a usual care control group. The results of this study will contribute to recommendations for future meditation interventions for chronic neck and shoulder pain.

**Trial registration:**

ISRCTN12146140. Registered on 20 August 2021.

**Supplementary Information:**

The online version contains supplementary material available at 10.1186/s13063-022-06873-x.

## Administrative information

The numbers in curly brackets in this protocol refer to SPIRIT checklist item numbers. The order of the items has been modified to group similar items.

**Table:** 

**Title {1}**	**Effectiveness of Anapana, Body scan and Metta meditation techniques on chronic neck and shoulder region pain and disability in adult patients in Sri Lanka.: study protocol for a cluster clinic-level parallel randomised controlled trial**.
**Trial registration {2a and 2b}.**	ISRCTN12146140 **Date of registration:** 20/08/2021
**Protocol version {3}**	Version 1. 08.03.2022
**Funding {4}**	Personal funds of the corresponding author
**Author details {5a}**	^1^Aranjan Lionel Karunanayake, ^2^Emma Solomon-Moore, ^3^Nikki Coghill ^1^ Department of Anatomy, Faculty of Medicine, University of Kelaniya, Sri Lanka & Department for Health, Faculty of Humanities and Social Sciences, University of Bath, Claverton Down, Bath, BA2 7AY, United Kingdom ^2,3^Department for Health, Faculty of Humanities and Social Sciences, University of Bath, Claverton Down, Bath, BA2 7AY, United Kingdom ^1^aranjan1368@gmail.com_;_ alk26@bath.ac.uk; ^2^es956@bath.ac.uk ^3^nc385@bath.ac.uk; **Corresponding Author** Aranjan Lionel Karunanayake
**Name and contact information for the trial sponsor {5b}**	University of Bath University of Bath, Claverton Down, Bath, BA2 7AY, United Kingdom
**Role of sponsor {5c}**	Providing educational support. Design, conduct and publication of the results of the study will be independent from the sponsor

## Introduction

### Background and rationale {6a}

People of all ages can be affected by neck pain [1]. Most patients with neck pain are also affected with shoulder region pain [2]. Due to difficulties in demarcating neck and shoulder region pain, many prevalence studies have considered these two regions together [3]. Bad neck posture while sleeping and carrying out activities of daily living are recognised as the main risk factors for neck pain [4]. In addition to physical factors, there is an association between chronic neck pain and psychosocial factors such as cognitive distress, anxiety and depression [1].

Techniques commonly used to manage neck and shoulder region pain include medications, physiotherapy, acupuncture, physical exercises and yoga [5]. Medications used in the management of musculoskeletal painful conditions are associated with an increased risk of allergic reactions, gastritis, ischemic heart disease, type 2 diabetes, cataract, nephropathy and osteoporosis [6]. Other treatment modalities such as physiotherapy, physical exercises and yoga can be associated with adverse effects, such as pain, swelling and soft tissue injuries [5], while techniques such as spinal manipulations, acupuncture and relaxation have not been associated with significant positive effects in the management of neck pain [7]. According to Monticone and colleagues [1], cognitive behavioural therapy has not been associated with significant positive improvements in levels of neck pain. Hence, there is scope for treatment methods, such as meditation, which are safe, cheap to implement, and easily accessible.

In meditation, the person learns to take control of their emotions to become the master of their own mind [8]. There are many different types of meditation techniques. Anapana, Body scan and Metta are meditation techniques that are practised worldwide [9]. Available research suggests how meditation can be useful for managing painful conditions [10]. A randomised controlled trial (RCT) conducted by Colgan and colleagues [10] reported that meditation training can improve an individual’s ability to observe and experience internal reactions to a stressor as they arise, with acceptance, and equanimity. In turn, this impartial receptiveness can reduce emotional reactivity to the stressor [10]. A clinical trial conducted to investigate the effect of meditation on 48 patients, aged 30–45 years, suffering from lower back pain, demonstrated that meditation is useful in reducing pain and enhancing physical and mental quality of life, compared to usual care [9]. In this study, patients practised three different meditation techniques for 90 min a day, for 8 weeks. However, because the intervention group practised three different meditation techniques, it was not possible to differentiate the effect of the individual meditation techniques on pain relief and quality of life. Asking patients to practise three different meditation techniques in order to manage their pain might be overly burdensome outside of a research environment. Being able to make recommendations for one technique in isolation is more likely to be adhered to by patients, as a treatment for their pain management. Similarly, a home-based RCT with 89 patients with chronic neck pain demonstrated that Jyoti meditation significantly reduced pain at rest as measured by numerical pain rating scale compared to a physical exercise programme [11]. Jyoti meditation involves practising a combination of four types of meditation techniques, such as concentration on breath, sound, the flame of a candle and loving kindness [11]. Since Jyoti meditation involves practising a variety of techniques in combination, it is difficult to assess the effectiveness of one type of meditation on pain reduction. In a systematic review of the effectiveness of meditation interventions for the treatment of chronic pain, Magoline et al. [12] stated that they were unable to identify any head-to-head trials comparing different meditation interventions with regard to pain and quality of life.

To date, studies that have investigated the effectiveness of meditation on pain relief and disability have been conducted in countries outside of Sri Lanka. Included interventions have mostly involved lengthy meditation times that may not be feasible for working adults, ranging from 20 min/day [13] to 120 min/day [9]. To date, studies that have involved meditation sessions of a shorter duration, i.e. < 15min, have not shown significant benefits with regard to physical, psychological and social disabilities [14]. However, a descriptive analytical study conducted by Kabat-Zinn et al. [15] involving 90 patients with a variety of conditions (low back pain, headache, migraine, facial pain, abdominal pain and neck and shoulder pain) demonstrated that practising meditation for 15 min/day for 10 weeks was associated with significant reductions in pain, negative body image, mood disturbances and pain-related drug utilisation. In this study, 33% patients were affected with low back pain and 27% patients were affected with headaches. Since the patients falling into other disease conditions such as neck pain, shoulder pain, facial pain and abdominal pain were small in number, the clinical effect of meditation on those conditions cannot accurately be determined.

Patients have expressed the importance of having a meditation programme that is brief, is useful for everyday living and can be easily incorporated into their daily routine [16]. Sri Lanka is a developing country and many patients in Sri Lanka prefer traditional treatment methods that are low in cost and have minimal or no adverse effects [17]. Therefore, meditation might be a suitable treatment method to fulfil this need. No studies to date have compared the effects of different types of meditation techniques on patients affected with chronic neck and shoulder region pain. Therefore, there is justified scope to find out which type of meditation is more effective in the management of chronic neck and shoulder region pain, alongside a programme that could easily fit into patients’ daily routines. Thus, the present study intends to compare the effectiveness of three different mindfulness meditation techniques (Anapana, Metta and Body scan) practised over a short duration (15 min) daily for 8 weeks, with each other and a usual care group, for adult patients affected with chronic neck and shoulder region pain.

### Objectives {7}

[SPIRIT guidance: specific objectives or hypotheses.]

- To compare the effectiveness of three different meditation techniques:
  - Anapana
  - Body scan
  - Metta

with a usual care control group and with each other, at 4, 8 and 12 weeks’ follow-up on patients’ perceived neck and shoulder region pain, range of movement at the neck and shoulder and changes in physical and social disability relating to activities of daily living, occupation and social activities with family and friends.

- Explore how patients in each intervention group feel about the effectiveness of meditation for their chronic neck and shoulder region pain.

### Trial design {8}

**Fig. 1:**
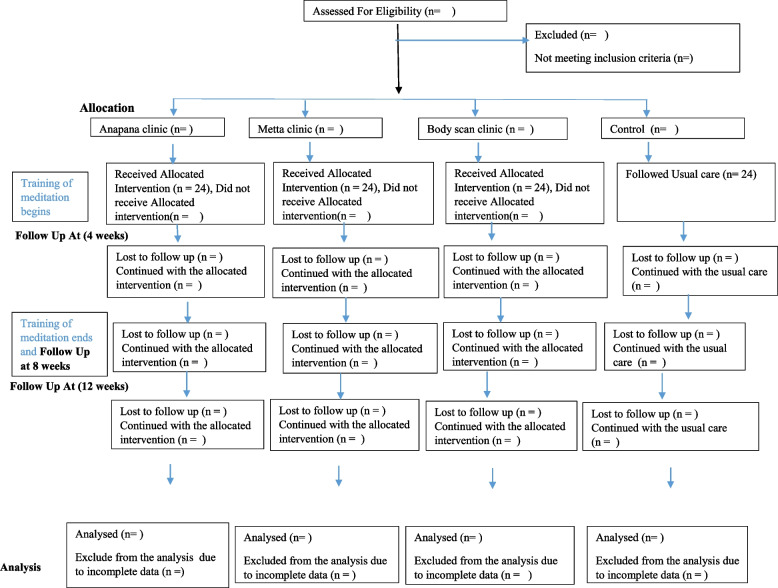
Flow chart to demonstrate the follow-up time periods

This is a four-arm parallel group single-centre trial with a superiority framework. The following flow chart (Fig. 1) provides an outline of the stages of the trial. This study will be conducted in-line with the CONSORT statement [18] (Fig. 1).

## Methods: participants, interventions and outcomes

### Study setting {9}

The study will be conducted in a rheumatology and family medicine clinic in Colombo, Sri Lanka.

### Eligibility criteria {10}

Study inclusion and exclusion criteria are described in Table 1.

**Table 1:** Inclusion and exclusion criteria

Inclusion criteria	Exclusion criteria
• Patients affected with chronic (> 12 weeks) neck and shoulder region pain attending the four clinics managed by the same physician and physiotherapist.	Patients affected with infections, inflammatory arthropathies, malignancies, Alzheimer’s disease, psychiatric conditions, severe depression and anxiety will be excluded.
• Patients affected with mechanical causes such as degenerative changes of the spine, muscle strain, ligament sprain.	Patients who take part in yoga and other meditation programmes.
• Patients who can understand and communicate in Sinhala.	Patients who are below the age of 18 years and above the age of 65 years.

### Who will take informed consent? {26a}

The corresponding author will collect written informed consent from the participants after explaining the details of the study to them.

### Additional consent provisions for collection and use of participant data and biological specimens {26b}

No biological specimens will be collected, and there are no ancillary studies planned. Therefore, no additional consent will be obtained.

## Interventions

### Explanation for the choice of comparators {6b}

The present study intends to compare the effectiveness of three different mindfulness meditation techniques (Anapana, Metta and Body scan) practised over a short duration (15 min) daily for 8 weeks, with each other and a usual care group, for patients affected with chronic neck and shoulder region pain. Each different meditation technique will represent a different intervention in the trial. These three techniques are commonly practised worldwide.

### Intervention description {11a}

This study consists of three intervention groups and a control group. Each intervention group will be practising a different meditation technique for 8 weeks in addition to usual care. The control group will follow only usual care. Usual care entails medication (e.g. non-steroidal anti-inflammatory) and physiotherapy. Participants in the meditation intervention will also receive the usual care.

The three different types of meditation techniques are:

- Anapana (concentration on breathing)
- Body scan (concentration on bodily sensations without reacting to them)
- Metta (concentration on loving kindness) [19]

In Anapana meditation, the attention is on ‘the breath’ and it focuses on ignoring any distractions that might break the chain of awareness on ‘the breath’. It helps to keep away thoughts of cravings, aversions, fantasies and illusions [8].

In Body scan meditation, the meditator observes bodily sensations in a systematic manner from head to feet and from feet to head; by not reacting to them, it helps the meditator to realise the impermanent nature of these sensations, and consequently, this will help the meditator to get rid of thoughts of cravings and aversions [8].

Practising Metta meditation helps to remove thoughts of anger and hatred from the mind and introduce thoughts of patience, friendship and love [20].

The meditation techniques will be taught to the patients by a meditation trainer who has more than 10 years of experience in teaching meditation. Meditation techniques will be taught on a weekly basis for 8 weeks. Other than the teaching session, participants will be provided with a leaflet giving them instructions on the technique. Each session will include five patients and last for 30–45 min [9]. In between weekly training sessions, patients in the intervention groups will be requested to practise the meditation technique that was taught to them, for 15 min each day. They will be advised to only practise the meditation technique that was taught to them and not to practise any other technique. Participants will be requested and instructed to keep a logbook record of their daily meditation practice. Participants will also be requested to log the type, dose and amount of medication taken each day.

### Criteria for discontinuing or modifying allocated interventions {11b}

Participants will not be subjected to any pain or harmful interventions. Therefore, we do not feel that there will be a necessity to modify the allocated intervention.

### Strategies to improve adherence to interventions {11c}

Daily short message services (SMS) will be sent to participants reminding them to practise their allocated meditation and to complete their log book. Weekly face-to-face meditation training sessions with the meditation trainer will also be used to address any concerns or difficulties experienced by participants.

### Relevant concomitant care permitted or prohibited during the trial {11d}

Participants in the intervention groups will be asked not to take part in any meditation or yoga programmes other than their allocated meditation intervention. Participants in the usual care group will also be requested not to take part in any meditation programmes or yoga during the 8-week intervention period of the trial. Additionally, participants will be advised not to engage in treatment for their neck and shoulder region pain, other than from the physician or therapist in the registered clinic as part of the trial. Pharmacological treatments for pain, for example anti-inflammatory medications, will not be prohibited during the trial if they are part of usual care provided by the physician.

### Provisions for post-trial care {30}

No harmful procedures will be conducted during the trial. Therefore, it is unlikely that the participants will need post trial care. If by chance the participants need any care, they will be referred to the appropriate specialist.

### Outcomes {12}

Primary and secondary outcomes are stated in Table 2.

**Table 2:** Primary and secondary outcomes

Primary outcome	Secondary outcomes
Differences in changes in pain in the neck and shoulder region at 8 weeks’ follow-up.	(i) Differences in changes in pain in the neck and shoulder region at 4 and 12 weeks’ follow-up.
	(ii) Differences in changes in the pain free active range of movement in the neck and shoulder region at 4-, 8- and 12-week follow-up.
	(iii) Differences in changes in quality of life with regard to activities of daily living, occupation and social activities with family and friends at 4, 8 and 12 weeks’ follow-up.
	(iv) To determine how patients in the three intervention groups feel about the effects of meditation on pain, physical and social disability with regard to activities of daily living, occupation and social activities with family and friends at 8 weeks’ follow-up.

Participants will be requested to report the pain intensity ratings from the previous 24 h using the Numeric Pain Rating Scale (range 0–10) [21] (Table 3) corresponding to:

- Pain level when they wake up in the morning
- Least pain
- Worst pain
  While pain intensity was assessed three times across 24 h, the mean score across the three ratings will be used as the primary outcome.
  The pain intensity ratings need to be maintained daily.

**Table 3:** Methods of measurement of outcomes

Outcome measure	Method of measurement
Level of pain	Numeric Pain Rating Scale, rated on a 0–10 scale [21]
Physical disability	Oswestry Neck Disability questionnaire [22], Disability of Arm, Shoulder and Hand (DASH) questionnaire [23], questionnaire to assess details of pain and demographic data, and clinical assessment of active range of movement of neck and shoulder joint [5]
Quality of life	The SF-36 Questionnaire Short Form [24]
Feelings on pain, physical disability and psycho social disability	This will be assessed qualitatively using focus groups.

Differences in quality of life will be assessed using the validated SF-36 questionnaire [24] (Table 3).

### Participant timeline {13}

The duration of the trial is 12 weeks and the supervised intervention duration is 8 weeks. The participants will be assessed at baseline and 4, 8 and 12 weeks’ follow-up.

### Sample size {14}

Sample size was calculated using the WinPepi version 11.65 statistical software. Results from a study by Jeitler et al. [11] with patients with chronic neck pain were used to calculate the sample size. In sample size estimation, we used the standard deviation values of the intervention group (17.2) and the control groups (21.5) after 8 weeks of treatment as stated in the study done by Jeitler et al. [11]. According to this study, the baseline pain value (45.5 SD 23.3) in the intervention group reduced to (21.6 SD 17.2) after 8 weeks of treatment and the baseline pain value (43.8 SD 22) in the control group reduced to (37.7 SD 21.5) after 8 weeks of treatment. Therefore, we considered 17.8 as the minimally important clinical difference. Using these values and a power of 80%, ratio of 1:1 and a significance level of 5%, the sample size was calculated. According to the calculation, the total sample size was 76 and number of patients for each arm of the trial was 19. According to an RCT conducted by Rantonen et al. [25] involving three intervention groups and a control group, the dropout rate was 26%. If we use this 26% dropout rate, and to maintain a total sample of at least 76, we need to have at least 26 patients per each arm of the trial. No adjustments will be made for multiple testing.

### Recruitment {15}

All patients who are not currently being treated for neck and shoulder pain in this clinic who meet the eligibility criteria and who attend the clinic on one of the intervention clinic days will be notified about the research study by an independent person (nurse). Patients who show their willingness to participate will be provided more information about the project and their written consent will be obtained prior to recruitment.

## Assignment of interventions: allocation

### Sequence generation {16a}

This is a four-arm parallel-group trial carried out in a single medical centre which has four clinic days. Each clinic day will be allocated to an intervention arm randomly, using the lottery method, by a person independent of the trial team. The allocation sequence will be computer generated. After a period of 6 months, the clinic days will be reallocated to intervention arms using the lottery method.

### Concealment mechanism {16b}

Allocation concealment will be implemented using sealed opaque envelopes. The usual care givers (doctor and the physiotherapist) and the physiotherapist who will be assessing the outcomes will not be aware of the randomisation allocation.

### Implementation {16c}

The allocation sequence will be generated by an independent statistician. Participants will be assigned to intervention groups and the control group by a clinic nursing assistant according to the instructions by the independent statistician. Study implementation will be completed by the researchers.

## Assignment of interventions: blinding

### Who will be blinded {17a}

Participants will be informed about the four trial arms at the time of agreeing to give informed consent. They are also informed that allocation of the four trial arms will be decided on a random basis. Participants in one arm will not know about the details of interventions that are given to participants in other arms.

### Procedure for unblinding if needed {17b}

In this trial, no harmful procedures are introduced and adverse effects are unlikely. In the event that an adverse effect did occur, appropriate assistance will be provided by an expert in the field who is independent to the trial team. Therefore, unblinding will not be required in this trial.

## Data collection and management

### Plans for assessment and collection of outcomes {18a}

The outcomes will be assessed using validated questionnaires and physical examinations (Table 3).

A questionnaire to assess details of presenting complaint, demographic data and details of medical and surgical history will be administered by the lead researcher. Physical and social disabilities will be assessed by a trained physiotherapist using methods listed in Table 3. Participants’ perceptions of the effects of meditation on pain, physical disability and psycho-social disability will be assessed qualitatively using focus groups conducted by the lead researcher. Focus groups will be applicable only to a subset of participants, but we will take steps to ensure that the subset of participants is representative of the wider participant population (i.e. age, gender, age, pain rating).

The focus groups will be audio-recorded and transcribed verbatim. The transcriptions in Sinhala will then be translated into English.

Data collection forms can be obtained on request from the lead researcher.

The Oswestry Neck Disability questionnaire consists of 10 sections which include questions on pain intensity, personal care (washing, dressing, etc.), lifting objects, reading, headaches, concentration, work, driving, sleeping and recreation. Under each section, there are five responses graded from 0 to 5, whereby zero indicates no disability and five indicates the highest disability [22].

DASH consists of three sections which include questions on activities performed at home, work and during recreation. First section has thirty questions. Under each question, there are five responses graded from 0 to 5, where zero indicates no difficulty and five indicates extreme difficulty [23].

The SF-36 questionnaire has 11 sections which include questions on vitality, physical functioning, bodily pain, general health perceptions, physical role functioning, emotional role functioning, social role functioning and mental health. Under each section, the potential responses range from 1 to 6 with higher scores indicating less disability [24].

### Plans to promote participant retention and complete follow-up {18b}

Participants in the intervention groups will be taught meditation by an experienced meditation instructor during the first 8 weeks on a weekly basis. At the beginning of the study, participants will be educated on the nature and potential purpose of meditation, and that for any potential benefits of meditation to be realised, it is important that they practise their meditation on a regular basis for the required period of time. This intervention does not include any harmful or painful procedures; thus, adverse events are unlikely. Participants will be regularly monitored on a weekly basis, and if by any chance an adverse event does occur, they will be referred to the appropriate specialist.

### Data management {19} and Confidentiality {27}

Participants will be allocated a unique identification number at the beginning of the study. Participants will not be photographed or video-taped, but they will be audio recorded for focus groups. All identifiable data (names, addresses, contact details) and the main data will be encrypted and stored separately from each other in a secure folder that can only be accessed by the research team. A locked filing cabinet will be used to store non-digital data. The keys will be accessible only by the research team. Data will be protected for 10 years. The results published from this study will be in the form of data for the whole group. During the study, monthly checks will be conducted by the research team comparing the hard and soft copies to identify any missing data.

## Statistical methods

### Statistical methods for primary and secondary outcomes {20a}

Descriptive statistics (means, SDs, percentages, *T*-tests, chi-square tests) will be used to describe patient characteristics at baseline, differences between groups at baseline, and for all outcomes at each follow-up time point (4-, 8- and 12-week follow-up). The significance level that will be used is 5% (corresponding 95% confidence intervals will be presented) [26] and the SPSS statistical soft wear version 21 will be used in data analysis. If data is normally distributed, a two-way ANOVA will be used to explore any changes in the primary outcome between patients in the three intervention arms (Anapana, Body Scan and Metta) and the usual care control group between baseline and the 8-week follow-up. Similarly, any changes in secondary outcomes between the four arms (three intervention arms and the control arm) will be explored at 4- and 12-week follow-ups. If the data is not normally distributed, a nonparametric equivalent, such as Kruskal-Wallis, will be used.

Treatment effects (between group differences) will be presented with corresponding confidence intervals.

Multivariate linear regression will be used to explore any effect of the independent variables of age, gender, level of income, level of education, changes in the amount of medication used and the weekly, mean meditation duration on the primary outcome at 8-week follow-up and in the secondary outcomes at 4- and 12-week follow-up. However, because they are not assessing differences between treatment arms, multivariate regression analyses will only be exploratory.

All focus groups will be audio-recorded and transcribed verbatim and translated to English [27]. For the qualitative data analysis, the literature recommends a scientific model be used [27]. To do this, the Richie and Spencer model will be used as it provides a sound analytical model [27]. ATLAS.ti will be used to manage the analysis due to its usefulness for coding.

### Interim analyses and stopping guidelines {21b}

Since there are no harmful procedures introduced and adverse effects are unlikely, stopping guidelines are not necessary for this study. Therefore, no interim analyses are planned.

### Methods for additional analyses (e.g. subgroup analyses) {20b}

This trial has four arms. Within each arm, there are no subgroups. Therefore, no additional analyses will be completed.

### Methods in analysis to handle protocol non-adherence and any statistical methods to handle missing data {20c}

Data analysis will be conducted on an intention to treat basis, even if there are cases of protocol non adherence. Study participants will be monitored on a weekly basis for protocol adherence. In between weekly periods, if the study participants need any advice, they have the opportunity to contact the meditation trainer. If any participants are not adhering to protocol, the reasons for not adhering to protocol will be discussed and advice will be provided on how to overcome any difficulties the participants are experiencing in following the protocol. It is not anticipated that there will be much missing data, due to outcome data being collected at patients’ clinic appointments; therefore, multiple imputation of missing data is not warranted. Steps will be taken to understand if data is missing at random, missing completely at random, or missing not at random, and appropriate statistical methods will then be used to handle missing data.

### Plans to give access to the full protocol, participant-level data and statistical code {31c}

When the trial results are published, an anonymised version of the dataset will also be provided as a supplementary file. Confidential data related to the trial participants will not be provided.

## Oversight and monitoring

### Composition of the coordinating centre and trial steering committee {5d}

The trial will be handled by the three researchers who will meet once every 2 weeks to assess how the trial is running. In addition, there will be a physician and physiotherapist involved in the routine treatment of patients. The meditating instructor will train the participants in the meditation techniques, while another therapist will assess the outcomes. Allocating patients to usual care control and intervention arms and registering patients will be carried out by a nursing assistant. Data entry will be conducted by a research assistant.

### Composition of the data monitoring committee, its role and reporting structure {21a}

Data monitoring will be conducted by the research team on a monthly basis.

### Adverse event reporting and harms {22}

The interventions do not include any harmful procedures. Therefore, adverse events are unlikely. If due to a rare chance an adverse effect occurs, it will be notified to the ISRCTN trial registry and also to the University of Bath Ethics committee.

### Frequency and plans for auditing trial conduct {23}

There are no harmful procedures introduced in this trial and adverse effects are unlikely. Therefore, there is no formal data monitoring committee. However, at the time that outcome data are assessed, a clinician who is independent of the trial team will monitor data analyses.

### Plans for communicating important protocol amendments to relevant parties (e.g. trial participants, ethical committees) {25}

At present, we do not feel any protocol amendments are required. However, if any important protocol amendments are done, it will be notified to the University of Bath Research Ethics Approval Committee for Health, ISRCTN trial registry and the participants.

### Dissemination plans {31a}

We intend to publish the trial results in peer-reviewed scientific journals and present these at scientific conferences. There are no publication restrictions.

## Discussion

The prevalence of work-related complaints of neck and shoulder among office workers in Sri Lanka is high (63.6%) [28], and comparable to the prevalence in developed countries (30–70%) [2, 29]. Most cases of neck and shoulder region pain are due to poor posture while at work [28]. In Sri Lanka, the retirement age is 65 years. Therefore, most patients affected with neck and shoulder region pain in Sri Lanka due to posture-related causes can be detected under the age of 65 years. So, exclusion of participants over the age of 65 years is unlikely to affect the generalisability of the data. Sri Lankan patients, similar to patients in other countries, prefer treatments that are low cost, with less side effects, and are easy to comply with [17].

Meditation has minimal or no negative side effects and the effect of meditation on easing pain and disability has not been studied in Sri Lanka. Comparing three meditation techniques with a usual care control group and with each other on pain, physical disability and psycho-social disability has not been described in previous literature. Therefore, conducting this study will contribute toward filling gaps in knowledge in this area of research. It may also evidence a treatment technique which is low-cost and less time consuming for managing patients who are affected with chronic neck and shoulder region pain in Sri Lanka. As a future study, to assess the robustness of findings, additional populations may be used.

### Study limitations

This is a four-arm parallel clinic-level randomised controlled trial. There can be issues related to clustering effects and allocation concealment. Therefore, allocation to a certain clinic day will be based on availability of sessions rather than patient characteristics, and all patients attending sessions on the relevant days will be systematically invited into the study. The similarity of study participants between intervention groups at baseline will be assessed.

In a randomised controlled trial, patients (or in this case clinic days) are randomly allocated to intervention or control arms. Therefore, patients may receive a meditation intervention that may not be the ideal technique for them personally. In this study, patients will be asked to meditate for at least 15 min/day on their own at home and record the practice duration in a logbook. Therefore, it will not be definitively possible to assess whether the meditation is actually performed or to assess the quality of meditation practised. Other limitations of the study is that analyses may be affected by imbalances between groups at baseline and the limited number of patients in each arm of the trial.

In the present study, expected loss to follow-up is high and potentially puts the study at risk of bias. To reduce dropouts in our study, the patients will be taught about the benefits of regular meditation prior to teaching them meditation. If patients experience any difficulty during their daily meditation practice, they have the facility to discuss them with the meditation instructor

### Study strengths

To evaluate the effect of an intervention over and above usual care, a randomised controlled trial is accepted as being the most rigorous study design [30]. The process of randomisation maximises the external validity of findings [30]. In many randomised controlled trials, interventions are compared with a control group but not with each other. In this clinic-based trial, the effect of three meditation interventions will be compared with a usual care control group, and with each other. To prevent patients discussing their group allocation with each other, patients are randomised to groups by clinic days rather than within clinics, making this a four-arm parallel trial. An additional strength of the study is that all the patents in the four groups will be treated by the same physician, physiotherapist and the meditation instructor. The study outcomes will also be assessed by the same person, who will be blinded to group allocation.

In addition to the quantitative component, this study includes a qualitative component to explore the experiences of patients receiving the three meditation interventions and how they feel the intervention has affected their chronic neck and shoulder region pain. Many randomised controlled trials that have been conducted to investigate the effects of meditation on pain management have only used quantitative measures to evaluate the intervention. Since meditation is a cost effective and a risk-free treatment technique this treatment method may be more acceptable for people in Sri Lanka compared to treatment methods such as medication and physiotherapy.

## Trial status

Initial Protocol version numbers were 01 and date 08.03.2022; 02 and date 10.08.22.

Amended Protocol version number is 03 and date 21.09.2022.

At the time this protocol paper was submitted, recruitment had not begun due to the COVID-19 pandemic situation in Sri Lanka. We anticipate that it will take 1 year to recruit and an additional 3 months to complete the monitoring. By the time we received reviewer comments on the manuscript, recruitment had started (recruitment start date: 18/04/2022).

## Supplementary Information

**Additional file 1.**

## Data Availability

The data will be accessible only by the three authors. On completion of the study, data will be stored in University of Bath Data Archive.

## Abbreviations

DASH: Disability Arm, Shoulder and Hand
SF-36: Short Form 36
