# Supplementary material for: Effectiveness of Anapana, Body scan and Metta meditation techniques on chronic neck and shoulder region pain and disability in adult patients in Sri Lanka: study protocol for a cluster clinic-level randomised controlled trial
Source: Trials. 2022 Nov 15;23:940. doi: 10.1186/s13063-022-06873-x (PMC9667001; doi:10.1186/s13063-022-06873-x)
Supplement: Supplementary file 1 — Additional file 1. [file 13063_2022_6873_MOESM1_ESM.docx]

**Consent form for participants in the intervention groups**

Researcher: Dr. Aranjan Lionel Karunanayake (Department of Health, Faculty of Humanities and Social Sciences, University of Bath; Email: – [alk26@bath.ac.uk](mailto:alk26@bath.ac.uk)

(Supervisors – Dr. Nikki Coghill, Department of Health, Faculty of Humanities and Social Sciences, University of Bath, Email – [N.Coghill@bath.ac.uk](mailto:N.Coghill@bath.ac.uk); Dr Emma Solomon-Moore, Department of Health, Faculty of Humanities and Social Sciences, University of Bath, [E.Solomon-Moore@bath.ac.uk](mailto:E.Solomon-Moore@bath.ac.uk))

**Please initial box if you agree with the statement**

| 1 | I have been provided with information explaining what participation in this project involves |  |
| --- | --- | --- |
| 2 | I have had an opportunity to ask questions and discuss this project |  |
| 3 | I have received satisfactory answers to all questions I have asked. |  |
| 4 | I have received enough information about the project to make a decision about my participation. |  |
| 5 | I understand that I am free to withdraw my consent to participate in the project at any time without having to give a reason for withdrawing. |  |
| 6 | I understand that I am free to withdraw my data within two weeks of my participation. |  |
| 7 | I understand the nature and purpose of the procedures involved in this project (providing answers to questionnaires, answering questions while in a group, measurements of my neck and shoulder movements, practicing a daily meditation technique for 15 minutes, keeping a record of (amount of meditation done per day, amount of medication taken/day, recording the pain intensity three times/day), attending to eight weeks of weekly meditation training sessions. I understand that I will be reviewed at baseline and thereafter, at four, eight and twelve weeks follow up at the clinic. These have been communicated to me on the information sheet accompanying this form. |  |
| 8 | I understand and acknowledge that the investigation is designed to promote scientific knowledge and that the University of Bath may use the data collected for this study in future research project(s), but that the conditions on this form under which I have provided the data will still apply. |  |
| 9 | I understand the data I provide will be treated as confidential, and that on completion of the project my name or other identifying information will not be disclosed in any presentation or publication of the research. |  |
| 10 | I agree to the University of Bath keeping and processing the data that I provide during the course of this study and my consent is conditional upon the university complying with its duties and obligations under the data Protection Act. |  |
| 11 | I hereby fully and freely consent to my participation in this project. |  |

Participant’s signature: _____________________________________ Date: ________________

Participant name in BLOCK Letters: _____________________________________

Researcher’s signature: ___ __ Date: ________________

Researcher name in BLOCK Letters: _----------------------------------------------

If you have any concerns or complaints related to your participation in this project please direct them to the Chair of the Research Ethics Approval Committee for Health, Dr James Betts ([j.betts@bath.ac.uk](mailto:j.betts@bath.ac.uk), 01225 383448)

Researcher: Dr. Aranjan Lionel Karunanayake (Department of Health, Faculty of Humanities and Social Sciences, University of Bath; Email: – [alk26@bath.ac.uk](mailto:alk26@bath.ac.uk)

Supervisors: Dr. Nikki Coghill, Dr. Emma Solomon-Moore

Contact details of Supervisors:

Department of Health, Faculty of Humanities and Social Sciences, University of Bath, [N.Coghill@bath.ac.uk](mailto:N.Coghill@bath.ac.uk)

Department of Health, Faculty of Humanities and Social Sciences, University of Bath, [E.Solomon-Moore@bath.ac.uk](mailto:E.Solomon-Moore@bath.ac.uk)

**Consent form for participants in the usual care group**

| Researcher: Dr. Aranjan Lionel Karunanayake (Department of Health, Faculty of Humanities and Social Sciences, University of Bath; Email: – [alk26@bath.ac.uk](mailto:alk26@bath.ac.uk);  (Supervisors – Dr. Nikki Coghill, Department of Health, Faculty of Humanities and Social Sciences, University of Bath, Email – [N.Coghill@bath.ac.uk](mailto:N.Coghill@bath.ac.uk); Dr Emma Solomon-Moore, Department of Health, Faculty of Humanities and Social Sciences, University of Bath, [E.Solomon-Moore@bath.ac.uk](mailto:E.Solomon-Moore@bath.ac.uk)) |
| --- |

**Please initial box if you agree with the statement**

| 1 | I have been provided with information explaining what participation in this project involves |  |
| --- | --- | --- |
| 2 | I have had an opportunity to ask questions and discuss this project |  |
| 3 | I have received satisfactory answers to all questions I have asked. |  |
| 4 | I have received enough information about the project to make a decision about my participation. |  |
| 5 | I understand that I am free to withdraw my consent to participate in the project at any time without having to give a reason for withdrawing. |  |
| 6 | I understand that I am free to withdraw my data within two weeks of my participation. |  |
| 7 | I understand the nature and purpose of the procedures involved in this project (providing answers to questionnaires, measurements of my neck and shoulder movements, keeping a record of (amount of medication taken/day, recording the pain intensity three times/day), I understand that I will be reviewed at baseline and thereafter, at four, eight and twelve weeks follow up at the clinic. These have been communicated to me on the information sheet accompanying this form. |  |
| 8 | I understand and acknowledge that the investigation is designed to promote scientific knowledge and that the University of Bath may use the data collected for this study in future research project(s), but that the conditions on this form under which I have provided the data will still apply. |  |
| 9 | I understand the data I provide will be treated as confidential, and that on completion of the project my name or other identifying information will not be disclosed in any presentation or publication of the research. |  |
| 10 | I agree to the University of Bath keeping and processing the data that I provide during the course of this study and my consent is conditional upon the university complying with its duties and obligations under the data Protection Act. |  |
| 11 | I hereby fully and freely consent to my participation in this project. |  |

Participant’s signature: ______________________________ Date: ________________

Participant name in BLOCK Letters: _____________________________________

Researcher’s signature: ___ __ Date: ________________

Researcher name in BLOCK Letters: _----------------------------------------------

If you have any concerns or complaints related to your participation in this project please direct them to the Chair of the Research Ethics Approval Committee for Health, Dr James Betts ([j.betts@bath.ac.uk](mailto:j.betts@bath.ac.uk), 01225 383448)

Researcher – Dr. Aranjan Lionel Karunanayake

Contact details: (Department of Health, Faculty of Humanities and Social Sciences, University of Bath, Email – [alk26@bath.ac.uk](mailto:alk26@bath.ac.uk);

Supervisors: Dr. Nikki Coghill, Dr. Emma Solomon-Moore

Contact details of Supervisors:

Department of Health, Faculty of Humanities and Social Sciences, University of Bath, [N.Coghill@bath.ac.uk](mailto:N.Coghill@bath.ac.uk)

Department of Health, Faculty of Humanities and Social Sciences, University of Bath, [E.Solomon-Moore@bath.ac.uk](mailto:E.Solomon-Moore@bath.ac.uk)
